# Dialogue: Validation of eight endotypes of lupus based on whole-blood RNA profiles

**DOI:** 10.1136/lupus-2025-001628

**Published:** 2025-07-21

**Authors:** Lina-Marcela Diaz-Gallo

**Affiliations:** 1 Division of Rheumatology, Department of Medicine Solna and Center for Molecular Medicine, Karolinska Institute, Stockholm, Sweden

**Keywords:** Lupus Erythematosus, Systemic, Inflammation, Information Science

 Effectively addressing the heterogeneity of SLE holds great promise for enhancing diagnosis and therapeutic strategies. This approach involves identifying distinct molecular signatures of SLE that can be useful in developing personalised diagnostic tools that account for individual variation, tailoring treatment plans, designing models for monitoring disease activity and even considering disease prevention. An increasing number of studies have contributed to this effort. Typically, each study uses a specific type of data—such as transcriptomics^1^,^3^ or autoantibody profile—to identify distinct SLE patterns, which are then tested for enrichment in clinical manifestations, disease activity or long-term outcomes. Among these strategies, the recently published validation study by Hubbard *et al*^1^ and its methodological predecessor^2^ focused on leveraging transcriptomic data to define SLE endotypes.

In this follow-up study,^1^ researchers used their earlier framework to categorise individuals with SLE into eight molecular subtypes.^2^ Using RNA sequencing data from 101 individuals enrolled in three separate clinical trials,^1^ the authors successfully applied their previously developed random forest model.^2^ This is a type of supervised machine learning—a method that teaches computers to recognise patterns from labelled examples and make classifications on unseen data. In this case, the model used the expression of gene sets to assign each patient to one of the eight predefined endotypes. Their results indicate a categorisation of the studied individuals into a transcriptome gradient that spans from endotypes closer to health to those reflecting more disease-related disturbances. Although differences in clinical features among the endotypes were modest, certain characteristics appeared more frequently in specific subgroups, particularly when comparisons were made between endotype pairs. An interesting relationship described by Hubbard *et al*^1^ is the enrichment of specific genetic ancestries within various transcriptome endotypes, which is related to a transcriptome - genetic ancestry - autoantibody positivity relationship previously described in the context of SLE by the same research group^4^ and used to define the gene sets for their SLE classification models. These findings echo other observations, where individuals of African ancestry tend to cluster in autoantibody-defined groups characterised by higher disease activity.^5^ It will be valuable to determine how much of that relationship is attributable to genetic risk factors or other components, such as environmental conditions rooted in social disparities.

A strength of Hubbard *et al*’s study^1^ is their use of the same method to analyse participants’ transcriptome (RNA sequencing), which helps reduce technical differences and potential confounders, often a limitation of this type of approach. Their findings support the value of transcriptome data for biological understanding and clinical applications, aligning with results from similar studies by other groups.^3^ Notably, these studies^1^,^3^ demonstrate how bioinformatics can reveal essential insights from various clinical trials and offer a method for combining diverse datasets. To maximise impact, it is critical to clearly communicate the studies—especially complex bioinformatics aspects—so rheumatologists and other researchers can grasp and apply the main findings.

There is still room to improve how studies are designed and carried out to understand the complexity of SLE. For instance, it is challenging to establish the influence of demographic, environmental and disease variables in the data used to subgroup individuals with SLE, such as whole blood transcriptomics, as in the referred studies,^1^,^3^ or autoantibody profiles in other approaches,^5 6^ which can affect the observations and conclusions. It is similar to the egg and chicken dilemma: are the disturbances in the expression of immune-related genes or specific autoantibody profiles a consequence of demographic and clinical variables, or vice versa? These challenges could be overcome by addressing appropriate sampling time points, implementing longitudinal designs, ensuring a broad and deep data collection and applying appropriate analyses. For example, studying individuals at risk of developing SLE and collecting data on immunological markers, omics, environmental factors and clinical variables over several years of follow-up will enable us to dissect the causal processes, which in turn will facilitate the development of disease prevention and prediction strategies.

Several approaches exist for identifying clinically meaningful SLE subgroups, all of which rely on the quality and breadth of input data. Improving data collection and overall study design requires collaboration among multidisciplinary experts, as well as the public and private sectors. Finally, as a research community, we should ensure that studies are comparable and independently validated. There is a tendency to overlook replication studies, which can undermine the value of reproducibility, a fundamental scientific principle.
